# Delayed Diagnosis of Hereditary Transthyretin Cardiac Amyloidosis Presenting with Autonomic Neuropathy: A Case Report

**DOI:** 10.3390/jcdd13100523

**Published:** 2026-10-16

**Authors:** Bingjie Song, Qijun Zhang, Lei Tao, Yingchun Bao, Chen Luo

**Affiliations:** 1 Cardiovascular Department, The Affiliated People’s Hospital of Ningbo University, Ningbo 315100, China; rmzhangqijun@nbu.edu.cn (Q.Z.); 15805841602@163.com (Y.B.); 15990256326@163.com (C.L.); 2 Cardiovascular Department, Beilun People’s Hospital, Ningbo 315800, China; 15868097668@163.com

**Keywords:** transthyretin protein, hypotension, hereditary transthyretin amyloidosis, autonomic neuropathy, diagnostic delay

## Abstract

**Background:** As a rare disease, cardiac amyloidosis (CA) has seen a deepening of epidemiological understanding, driven by advances in imaging and genetic testing, alongside drug development. However, the identification of suspected patients in the early stages remains inadequate. **Case Presentation:** Our 64-year-old patient carrying the rare transthyretin variant p.Gly67Val (legacy nomenclature Gly47Val) originally presented with lower limb numbness, resulting in prolonged follow-up at neurology outpatient clinics. Subsequently, unexplained hypotension led to hospital admission. The atypical early symptoms and inaccessibility of relevant tests delayed diagnosis and treatment. **Discussion:** As illustrated by this case, patients with CA referral to a specialist for further diagnosis and treatment typically occur when heart failure and conduction abnormalities have developed, a stage at which treatment outcomes are substantially limited. The p.Gly67Val variant ATTR-CA has been reported in about 20 carriers worldwide, with phenotypes ranging from neuropathy-dominant to cardiac-dominant disease; this case extends the emerging mixed phenotype of this spectrum. **Conclusions:** This case highlights the importance of recognizing extracardiac manifestations to reduce diagnostic delay in hereditary transthyretin cardiac amyloidosis.

## 1. Introduction

Cardiac amyloidosis (CA) is an underdiagnosed infiltrative cardiomyopathy. This condition arises from the deposition of misfolded proteins in the extracellular space, where they aggregate into amyloid fibres [1]. The two most common types are immunoglobulin light chain (AL) CA and transthyretin (TTR) CA, with the latter caused by misfolded monomers or oligomers of wild-type (ATTRwt) or variant TTR (ATTRv) [2]. ATTRv is inherited in an autosomal dominant pattern and is caused by mutations in the TTR gene, which is located on chromosome 18. To date, over 130 distinct mutations have been identified [1]. According to reports from the United States, the prevalence of ATTR-CA (unclassified variant or wild-type) in the general population is approximately 6.1 per million [3]. Concurrently, a large-scale study encompassing 205,545 cases determined that the prevalence of ATTR-CA among patients hospitalised for heart failure was 0.31% [4]. The prevalence increases with age. A study conducted at 20 hospitals in Spain examined 387 consecutive outpatients and inpatients aged 50 years or older with heart failure with preserved ejection fraction (HFpEF) and left ventricular hypertrophy (LVH) of at least 12 mm. The results showed that the overall prevalence of ATTR was 16.8%, with only 7% of cases being ATTRv-CA [5]. Asia lacks large-scale cohort studies, necessitating more case reporting. The diagnosis and treatment of ATTR amyloidosis are of critical importance, as the condition can pose a life-threatening risk to patients, with an expected survival of only 3 to 15 years after the onset of symptoms [6].

Advances in imaging technology have facilitated the transition from invasive endomyocardial biopsy to noninvasive examination for the diagnosis of CA [7]. Especially technetium-99m pyrophosphate (^99m^Tc-PYP) imaging, which has been demonstrated to have a sensitivity of 91% and a specificity of 92% in the detection of ATTR cardiac amyloidosis through multicentre studies. The study demonstrates high sensitivity and specificity in distinguishing patients with ATTR cardiac amyloidosis (regardless of genotype) from those with AL-CA and patients with preserved ejection fraction non-amyloidotic heart failure. For patients with a high clinical suspicion of cardiac amyloidosis, ^99m^Tc-PYP serves as a diagnostic and prognostic assessment tool [8]. Nevertheless, due to limitations in available resources, national healthcare systems and clinicians are confronted with a challenging dilemma concerning the cost-effectiveness of conducting screening and treatment for ATTR. As demonstrated in the research conducted by Lau et al. within the context of the U.S. healthcare system, the incremental cost per additional quality-adjusted life year (QALY) over an estimated median survival of 7.5 years has been determined to be $919,509 [9,10]. Furthermore, the accessibility of these diagnostic procedures and pharmaceutical interventions is not uniform across all medical facilities, resulting in delays in the process of diagnosis and treatment.

## 2. Case Presentation 

### 2.1. Patient Information

We report a 64-year-old male with no previous history of hypertension or diabetes, who first presented to our department on 27 August 2023, with a chief complaint of recurrent dizziness accompanied by unexplained orthostatic hypotension with blood pressure of approximately 85/47 mmHg for a period of two months. The interval between the initial presentation and the diagnosis of ATTR-CA was approximately two years (Figure 1).

### 2.2. Clinical Findings

At presentation, orthostatic blood pressure was assessed: after 5 min of supine rest, blood pressure was 106/62 mmHg, which fell to 94/55 mmHg and 90/50 mmHg at 1 and 3 min of standing, respectively, meeting the criteria for orthostatic hypotension (a drop of ≥20 mmHg systolic and/or ≥10 mmHg diastolic). The electrocardiogram (ECG) (Figure 2A; Table S1) showed left anterior hemiblock (LAH) and pseudoinfarction (V1–V3 abnormal Q waves), without typical atrioventricular block (PR interval of 188 ms) and low voltage in limb leads. A transthoracic echocardiogram (TTE) (Figure 3A,B) revealed preserved ejection fraction (EF 60%), interventricular septal thickening (IVST, approximately 13–16 mm, predominantly anterior septal), and moderate aortic valve regurgitation. The latter was precisely what we initially considered the cause of the patient’s hypotension. At that time (Table S2), the patient’s brain natriuretic peptide (BNP) level was 115 pg/mL (reference range <100 pg/mL), the troponin I level was 0.0372 ng/mL (reference range 0–0.0175 ng/mL), and their creatinine level was 62 μmol/L (reference range 57–111 μmol/L). Due to the abnormal Q waves and mildly elevated troponin, a coronary angiography was performed, which revealed no significant abnormalities, thereby ruling out the possibility of ischemic cardiomyopathy. No significant abnormalities were observed in the expression of immunoglobulins G, A, and M (IgG, IgA, IgM), as well as complement 4 (C4). Complement 3 (C3) demonstrated a slight decrease (Table S3); however, this isolated mild reduction has limited clinical significance. Furthermore, during the initial clinical evaluation, the patient did not exhibit signs of anemia, renal insufficiency, macroglossia, or periorbital ecchymoses; therefore, clinical suspicion of AL-CA was low at that time, and further tests—such as serum free light chains (sFLC) and serum and urine immunofixation—were not performed to rule out AL-CA. This was a limitation on our part at the time. At the same time, the patient had no history of hypertension, which makes it unlikely that the hypertrophy was caused by hypertensive heart disease. In consideration of the patient’s ameliorated symptoms and the logistical challenges associated with conducting additional testing during that period, the underlying cause of the ventricular septal hypertrophy could not be unequivocally ascertained.

It is noteworthy that the patient initially presented with numbness in both lower limbs (the neuropathy has been staged using the Portuguese Peripheral Neuropathy Disability (PND) scale as stage I, based on clinical examination) in June 2023, followed by recurrent diarrhoea in November 2023. The patient had undergone long-term follow-up in both neurology and gastroenterology. The patient was admitted to the gastroenterology department after suffering from recurrent diarrhoea for over a year in November 2024. At this point, the ECG (Figure 2B) revealed first-degree atrioventricular block (AVB, PR interval of 222 ms) and QT interval prolongation. The EF and IVST showed no significant change on TTE (Figure 3C,D), with enlargement of the left atrium and left ventricle compared to previous measurements (40 mm vs. 37 mm, 53 mm vs. 49 mm). The symptoms of peripheral and autonomic neuropathy described here align with those reported previously as common extracardiac manifestations of ATTRv-CA.

### 2.3. Diagnosis

Until March 2025, when the patient presented at our outpatient clinic for heart failure (NYHA functional class II–IIII; brain natriuretic peptide (BNP) 690 pg/mL, reference range <100 pg/mL), suspicion of cardiac amyloidosis was raised again based on the patient’s history, which included a previous diagnosis of myocardial hypertrophy, chronic diarrhoea, and numbness in both hands and feet. Repeat testing of immunoglobulins (IgG, IgA, IgM), complement (C3, C4), serum and urine total light chains, and serum immunofixation electrophoresis was performed (Table S3); in the context of the patient’s clinical presentation, these results were interpreted as making AL-CA unlikely, although formal exclusion by current diagnostic algorithms was not achieved. It was recommended that the patient undergo ^99m^Tc-PYP scintigraphy at another medical facility. The report, which met the noninvasive diagnostic criteria for ATTR-CA, was as follows: The semi-quantitative visual grading system for myocardial perfusion scored 3 points. The H/CL (heart to contralateral lung) ratio was 2.26 at 1.5 h (Figure 4A) and 2.03 at 3 h (Figure 4B). Abnormally increased radiotracer activity was shown by SPECT throughout the myocardium, with the greatest increase seen in the left ventricle (Figure 4C). Cardiac magnetic resonance imaging (CMR) showed diffuse subendocardial late gadolinium enhancement (LGE) involving the entire left ventricle, with a circumferential distribution affecting all myocardial segments (Figure 5). Based on CMR, in conjunction with positive ^99m^Tc-PYP scintigraphy, and in the absence of symptoms such as acroparesthesias, angiokeratomas, hypohidrosis, cornea verticillata, or renal involvement with proteinuria, Fabry disease was not suspected. Regrettably, GLA gene sequencing and α-galactosidase A activity assay were not performed; thus, formal exclusion of Fabry disease was not achieved. However, the overall phenotype was considered strongly atypical for this disorder. Furthermore, the patient was subjected to genetic testing for hereditary diseases, which revealed the transthyretin mutation (p.Gly67Val, legacy nomenclature G47V; NM_000371.4:c.200G>T; rs121918090) (shown in Figure S1). The p.Gly67Val variant is registered in ClinVar (https://www.ncbi.nlm.nih.gov/clinvar/; Variation ID 938206, accessed on 10 June 2025) with a consensus classification of pathogenic/likely pathogenic by three independent clinical laboratories (Labcorp/Invitae, SCV001378733.6; SCV004813562.2; Ambry Genetics, SCV002722875.2) based on ACMG/AMP criteria, with no conflicting interpretations. The variant is absent from gnomAD (PM2), has been reported in multiple unrelated patients with hATTR amyloidosis (PS4 [11,12,13,14]), and is supported by functional evidence demonstrating markedly reduced tetramer stability comparable to the established pathogenic L55P mutant (PS3 [15]). It is also curated in the dedicated amyloidosis mutation database (amyloidosismutations.com, accessed on 10 June 2025) with cardiac and neuropathic phenotypes. This mutation has been reported in other patients with ATTRv-CA [15]. But the mutation is extremely rare, with almost twenty cases reported worldwide. It exhibits significant phenotypic heterogeneity and is classified as a globally rare variant [13,15,16], with no evidence of racial clustering (shown in Table S3). In conjunction with the patient’s medical history, the diagnosis of ATTRv-CA has been confirmed.

### 2.4. Treatment and Outcome

Following confirmation of the diagnosis, the patient was initiated on oral treatment with 61 mg of tafamidis, a TTR stabilizer, once daily in June 2025. In December 2025, the patient presented with significant sinus bradycardia, intermittent first-degree AVB, and complete left bundle branch block (CLBBB). A dual-chamber permanent pacemaker (Abbott 2088-TC52) was implanted in December 2025, with leads placed in the right atrial appendage and left bundle branch area. During the post-discharge follow-up conducted every 2–4 weeks, the patient’s heart failure remained relatively well controlled at NYHA class II, with no limitation of daily activities. The patient’s most recent follow-up BNP level was 121.7 pg/mL in June 2026 (shown in Table S2). Persistent extracardiac manifestations included peripheral neurologic symptoms—including numbness in both lower limbs (PND stage I)—as well as autonomic symptoms such as chronic diarrhea and hypotension. The blood pressure stabilized in the range of 90–100/50–60 mmHg without symptomatic deterioration. Notably, there was no progression of extracardiac symptoms. The patient maintained an optimistic outlook, with preserved health-related quality of life.

## 3. Discussion

This case is informative for four reasons: (i) it expands the phenotypic spectrum of the rare p.Gly67Val variant; (ii) it documents the evolution of conduction disease from initial ECG finding to eventual pacemaker; (iii) it illustrates a 2-year diagnostic delay with identifiable missed opportunities, and demonstrates the need to integrate autonomic, gastrointestinal, neurological, and cardiac manifestations rather than evaluating each in isolation.

According to data from the Transthyretin Amyloidosis Outcomes Survey (THAOS), patients with ATTRv-CA more frequently exhibited cardiac involvement (100.0% vs. 88.5%), autonomic neuropathy (53.7% vs. 21.2%), sensory neuropathy (51.9% vs. 15.4%), motor neuropathy (11.1% vs. 1.9%), and gastrointestinal symptoms (31.5% vs. 0%) than those with ATTRwt-CA. All 238 patients initially enrolled who presented primarily with neurological symptoms (54.2% male, 76.3% white) had ATTRv-CA [17]. About the p.Gly67Val variant, since its first description in a Sri Lankan kindred with a mixed phenotype of peripheral and autonomic neuropathy, carpal tunnel syndrome, and cardiac involvement in 1993 [18], nearly 20 individually characterized carriers of the heterozygous TTR c.200G>T (p.Gly67Val; legacy nomenclature G47V) variant have been reported worldwide, yet the accumulated cases already reveal a remarkably broad phenotypic spectrum (Table S4) [13,15,16,19,20,21]. The earliest and most numerous reports came from Asia and were dominated by neurological disease: Asakura et al. [13] described a Japanese woman in her eighth decade with peripheral neuropathy, autonomic dysfunction manifested as refractory diarrhea, and coexisting cardiac involvement, and Suenaga et al. [19] reported two Japanese cases of G47V-associated familial amyloid polyneuropathy. In the multi-ethnic Malaysian cohort of Low et al. [16], two siblings carrying the identical variant both developed peripheral neuropathy with abnormal ECGs, but only one progressed to overt heart failure—an instructive example of intrafamilial phenotypic discordance. Similarly, the Chinese cases reported by Du et al. [21] presented with sensory-predominant polyneuropathy, autonomic dysfunction, and cardiac involvement. Cardiac-predominant disease, by contrast, has been documented more recently and appears to run a more aggressive course. He et al. [15] reported a 52-year-old Chinese man with early-onset ATTR-CA whose disease began with syncope from complete heart block requiring permanent pacing at approximately 48 years of age, followed by rapidly progressive heart failure with severely reduced ejection fraction and Perugini grade 3 PYP uptake, in the absence of peripheral neuropathy, carpal tunnel syndrome, or gastrointestinal symptoms; his mother had suffered sudden cardiac death within a year of pacemaker implantation, suggesting a malignant, conduction-system–predominant phenotype in that lineage. In this case, the patient had already experienced symptoms of autonomic and peripheral neuropathy, including hypotension, diarrhoea, and limb numbness, before the heart failure episode. Several observations from the reported cases are relevant to clinical practice. First, cardiac involvement is not a late complication but an integral feature of G67V disease: abnormal ECGs were present in both neurologically affected Malaysian siblings, and cardiac involvement accompanied neuropathy in the Sri Lankan, Japanese, and Chinese reports. Second, the age at onset spans at least three decades, from the forties in cardiac-predominant cases to the eighth decade in neuropathy-predominant cases, and no single organ phenotype predicts the variant’s behavior in an individual patient. Third, p.Gly67Val is one of four pathogenic substitutions at codon 47/67 of TTR—alongside Gly47Arg, Gly47Ala, and Gly47Glu—which together define a mutational hot spot; notably, Gly47Arg and Gly47Glu were associated predominantly with neuropathic or mixed phenotypes in European kindreds [22,23], whereas the available G47V data suggest a stronger cardiac predilection. This phenotypic heterogeneity, both between and within families carrying the same variant, argues that all identified carriers of p.Gly67Val should undergo systematic cardiac assessment—including ECG, Holter monitoring, echocardiography with strain imaging, and, where indicated, bone-tracer scintigraphy—regardless of the presenting complaint or neurological status, and that unexplained conduction disease in a young or middle-aged patient, particularly with a suggestive family history, should prompt consideration of hereditary transthyretin amyloidosis even in the absence of neuropathy.

In addition, we find that the changes in the electrocardiogram exhibited dynamic progression (Table S1), which perhaps guides us in assessing the progression of a patient’s disease. At the time of presentation with hypotension, the baseline ECG showed Q waves in leads V1–V3, as well as LAH. An ECG performed 13 months earlier showed sinus rhythm with no significant abnormalities. 18 months later, the patient’s ECG showed sinus bradycardia, first-degree AVB, and a prolonged QT interval. By month 24, CLBBB had developed. By month 28, significant sinus bradycardia had emerged, resulting in a diagnosis of sick sinus syndrome (SSS). QTc prolongation is a frequent finding in cardiac amyloidosis (reported in more than half of patients, without significant differences between ATTR and AL subtypes) and, in ATTR-CA, has been shown to correlate with markers of disease severity, including NYHA class, NAC stage, NT-proBNP, and troponin levels [24]. On our patients’ electrocardiograms, we can also observe that the QTc progressively lengthens as the disease progresses, and the ECG changes correspond to the progression of the disease. However, QTc prolongation is not specific to ATTR, and in patients with conduction disease it may largely reflect QRS widening rather than a primary repolarization abnormality, as the JT interval is often preserved [25]. Its interpretation in the present patient—who had LBBB/bradycardia—is therefore limited, and the machine-derived values (Bazett-corrected) are reported with the accompanying heart rates and QRS duration in Table S1.

For this patient, the period from the onset of symptoms to the development of heart failure was approximately 22 months; the confirmation of the underlying cause through noninvasive imaging, genetic testing, and monoclonal negativity testing took more than 24 months; and by 28 months, arrhythmias had developed that required the implantation of a pacemaker (shown in Figure 1). Based on previous literature and this case study, it can be observed that for most patients with the p.Gly67Val mutation, the initial symptoms are typically not cardiac in nature. These non-cardiac symptoms can mislead both patients and doctors, leading to a delay in diagnosis, meaning patients will not receive an accurate diagnosis or effective treatment in the early stages of the disease. Additionally, even though the TTE had already indicated heterogeneous myocardial hypertrophy during the patient’s initial hospitalization for hypotension, no further investigation into the mechanism or etiology was conducted due to the atypical nature of the patient’s symptoms and ECG findings, coupled with the fact that the ^99m^Tc-PYP technique was not available at the local hospital at that time. Data from the ATTR-ACT trial showed that the placebo group (177 patients with a 30-month follow-up) had an overall mortality rate of 42.9% (76 deaths). The mortality rate was significantly higher among ATTRv-CA patients (62.8%) than among ATTRwt-CA patients (36.6%) [26]. In the tafamidis group, the 30-month data of the ATTR-ACT trial indicate that all-cause mortality was reduced by 30% compared to the placebo group (HR 0.70, 95% CI 0.51–0.96) [27]. This further highlighted the importance of adopting a multidisciplinary, holistic approach to patient care when developing specialised expertise. Fostering greater interdisciplinary communication can help us to detect diseases earlier and establish standardised diagnostic and treatment protocols [28].

In retrospect, the initial presentation contained multiple red flags that should have prompted earlier consideration of CA.

Echocardiography: concentric LVH, disproportionate to the hypotension.

Autonomic: orthostatic hypotension and chronic diarrhea in a middle-aged man, unexplained by common causes.

Polyneuropathy: had a history of numbness in the lower extremities and was being followed up at the neurosurgery outpatient clinic, but the cause remains unknown.

None of these findings were integrated at the time; each was evaluated in isolation by the respective specialty, contributing to the 2-year delay to diagnosis.

Regarding the treatment, disease-modifying therapies for ATTR comprise two mechanistic classes: tetramer-stabilizing agents, which kinetically stabilize the TTR tetramer and prevent its dissociation into amyloidogenic monomers (tafamidis and acoramidis), and TTR gene-silencing agents, which suppress hepatic TTR synthesis (the small interfering RNAs patisiran and vutrisiran, and the antisense oligonucleotide eplontersen). Their availability in mainland China, however, remains uneven. Tafamidis has been approved since 2020 for both ATTR-PN and ATTR-CA and is reimbursed through the national dual-channel system, although with indication-specific restrictions (stage I ATTR-PN for the meglumine formulation; ATTR-CA for the free-acid formulation). In contrast, neither patisiran nor vutrisiran has been approved by the NMPA to date. Eplontersen was approved for ATTRv-PN in December 2025—the first gene-silencing agent available in China for this disease—but has not yet entered the reimbursement list. This regional landscape directly shaped the treatment of the present patient: tafamidis monotherapy was the only disease-modifying option that was simultaneously approved, evidence-supported for the cardiac phenotype, and financially accessible at the time of treatment initiation.

### 3.1. Strengths and Limitations

The strengths of the report were providing a relatively comprehensive account of a patient’s progression from peripheral neurologic and autonomic symptoms to heart failure and atrioventricular block. Furthermore, it provides a visual representation of the alterations observed in electrocardiograms and echocardiograms as the patient’s condition evolved. Additionally, it elucidates the utilisation of imaging and genetic sequencing techniques in the definitive diagnosis of the disease.

However, there are several limitations to this study. As an individual case report, our findings are inherently limited in generalizability. The diagnostic trajectory and clinical course described may not be representative of all patients with cardiac amyloidosis, particularly given the heterogeneity of disease presentation across ATTR subtypes. Multi-center cohort studies are needed to quantify the frequency and determinants of diagnostic delay at a population level. In the meantime, as a retrospective data collection method, the analysis of missed diagnostic opportunities relies on retrospective chart review, which is susceptible to incomplete documentation bias. We were unable to ascertain the precise clinical reasoning behind each prior clinical encounter, and some early disease manifestations may have been under-recorded. Prospective diagnostic pathway audits would provide more robust evidence for systemic improvement. In addition, cardiac biomarkers (BNP, hs-cTnI) were elevated (above the 99th percentile reference limit), consistent with the observed cardiac phenotype (shown in Table S2). But formal disease staging according to the NAC or Mayo systems was not possible, as these systems were validated using NT-proBNP and cardiac troponin T, which were not available at the institution; BNP and hs-cTnI cannot be substituted into these algorithms owing to the absence of validated thresholds. This represents a limitation of the present report [29].

### 3.2. Take-Away Lessons

We need to maintain a high index of suspicion for infiltrative cardiomyopathies in patients with extracardiac “red flags” such as carpal tunnel syndrome, lumbar spinal stenosis, and autonomic neuropathy—symptoms that often precede cardiac manifestations by years. As well, adopt a holistic, multidisciplinary diagnostic approach rather than an organ-centric perspective. Cardiac amyloidosis is a systemic disease; collaboration between cardiology, hematology, neurology, and pathology is essential for timely diagnosis and appropriate phenotyping (ATTR vs. AL). Additionally, optimize regional healthcare networks and facilitate timely referral to specialized amyloidosis centers when local expertise or advanced therapies (e.g., tafamidis, patisiran, vutrisiran, eplontersen) are unavailable. Diagnostic delay directly translates into irreversible organ dysfunction and lost therapeutic opportunity.

### 3.3. Patient Perspective

The patient described a prolonged diagnostic odyssey spanning almost 2 years, during which symptoms affecting multiple organ systems were evaluated in isolation by various specialists. He expressed frustration that no clinician had systematically integrated these disparate manifestations into a unified diagnostic framework until advanced cardiac dysfunction had already developed. The patient emphasized the emotional burden of diagnostic uncertainty and the profound impact of delayed recognition on therapeutic options and long-term prognosis. He voiced a strong desire that this case serve as a reminder to clinicians of the importance of holistic patient assessment and cross-specialty communication, particularly in the setting of multisystem disease presentations.

## 4. Conclusions

Cardiac amyloidosis is a systemic disease that affects multiple organs. It is most commonly diagnosed when symptoms of heart failure appear. However, early extracardiac manifestations, such as peripheral and autonomic neuropathy, should not be overlooked. Early diagnosis and treatment are closely linked to patient prognosis and life expectancy. Therefore, there is a need for greater awareness of the diagnosis and management of the disease across disciplines, as well as the promotion of appropriate diagnostic and therapeutic approaches.

## Figures and Tables

**Figure 1:**
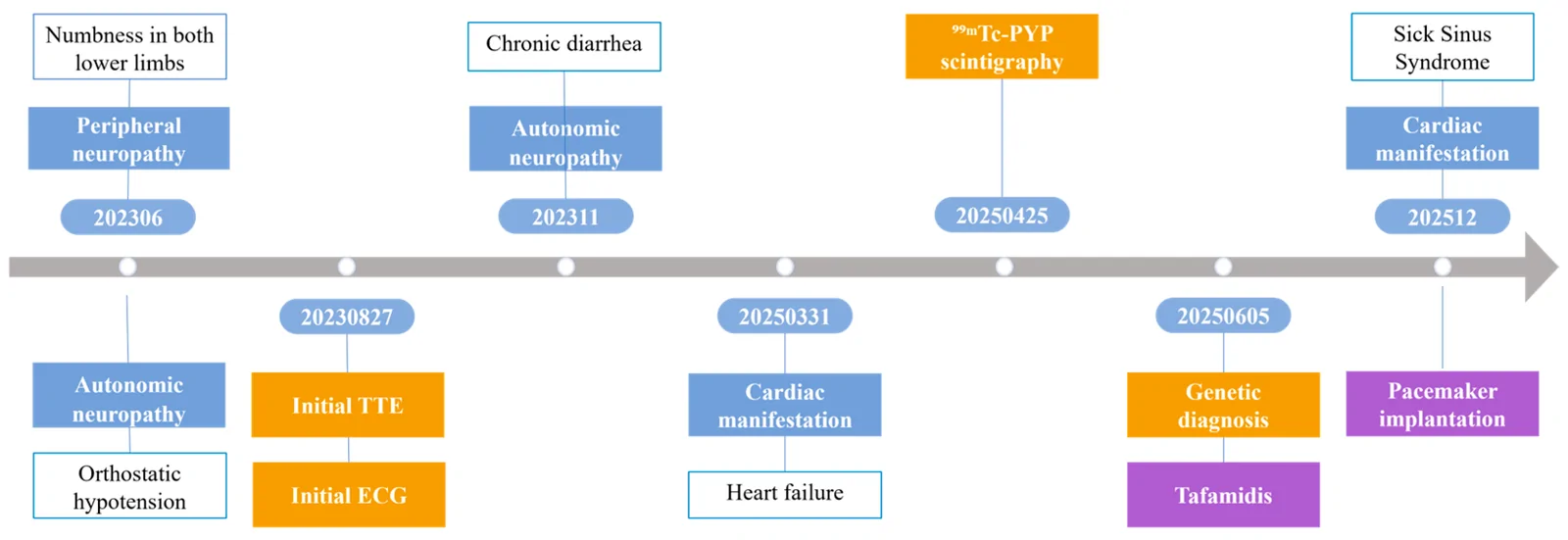
Clinical timeline of the patient. The blue boxes represent time and symptoms, the yellow boxes represent tests, and the purple boxes represent treatment.

**Figure 2:**
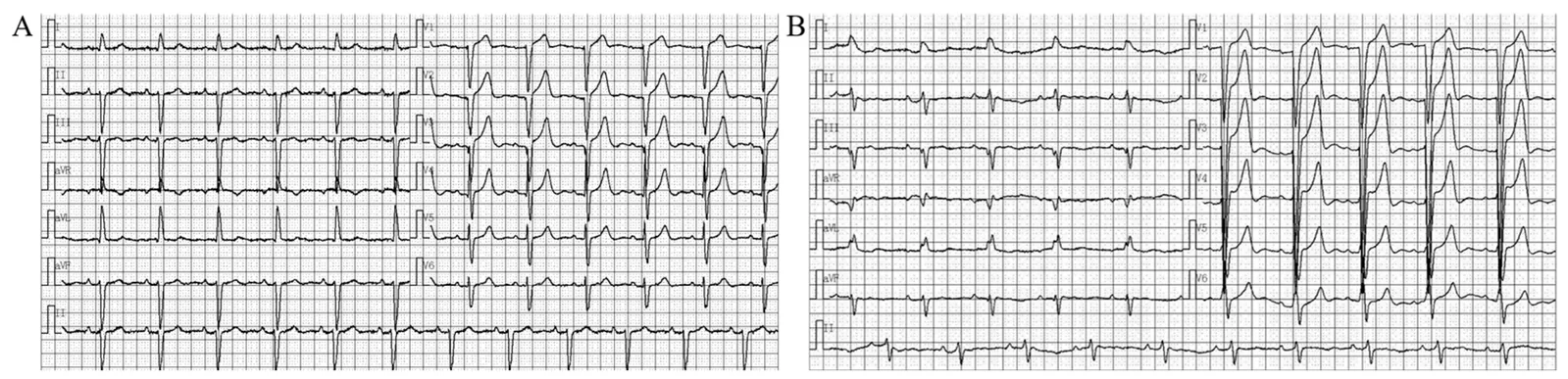
12-lead Electrocardiography (ECG) examinations (25 mm/s, 10 mm/mV). (**A**) shows an ECG from August 2023, while (**B**) shows one taken one year later, in December 2024.

**Figure 3:**
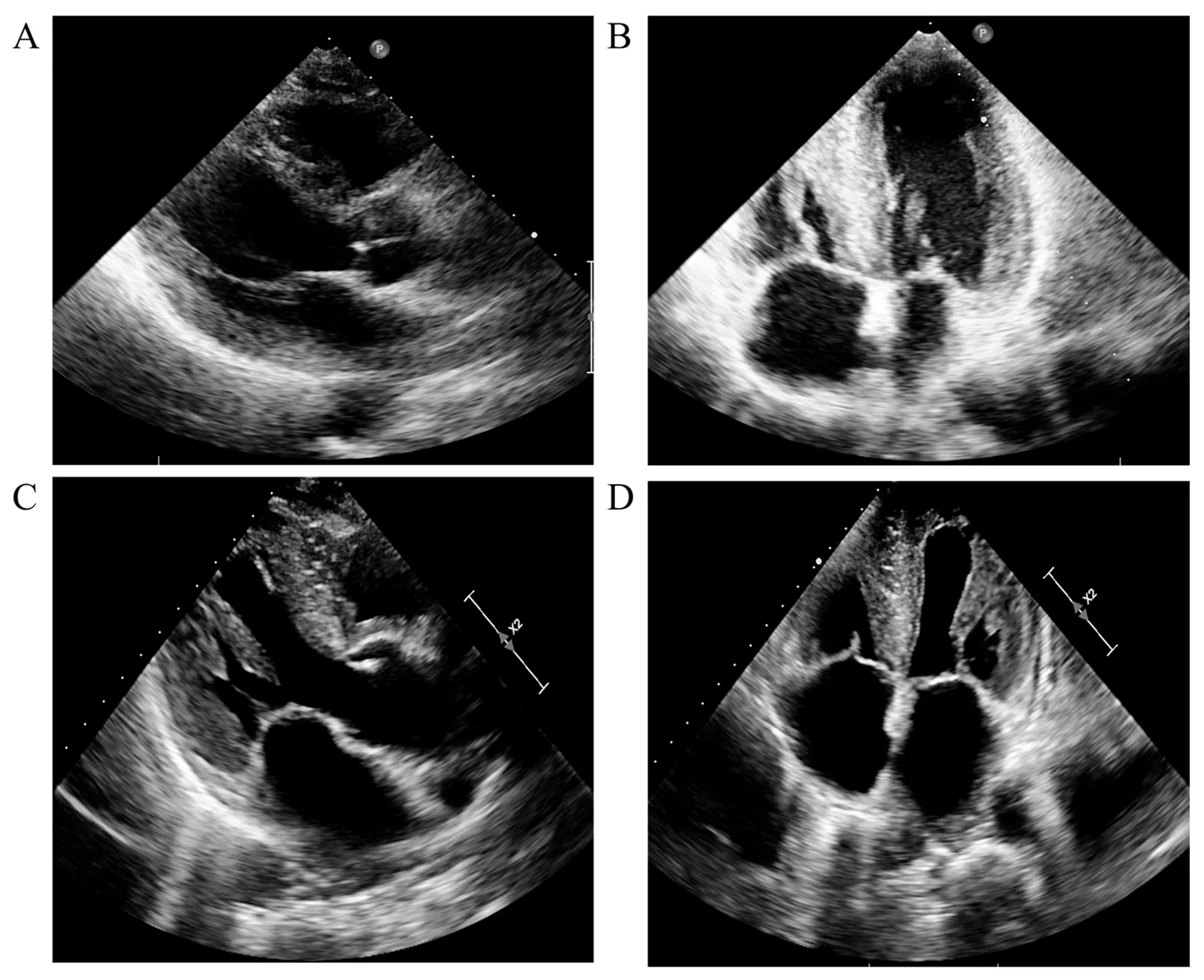
Echocardiographic findings. Parasternal long-axis views (**A**,**C**) and apical four-chamber views (**B**,**D**) demonstrate increased left ventricular wall thickness with a granular, sparkling appearance of the myocardium. Images (**A**,**B**) were obtained in 2023, while images (**C**,**D**) were obtained one year later. The interventricular septum exhibited persistent thickening at the subsequent follow-up, with no substantial alteration in thickness in comparison with the preceding study. Conversely, the left atrium exhibited an increase in size on subsequent imaging relative to the initial examination.

**Figure 4:**
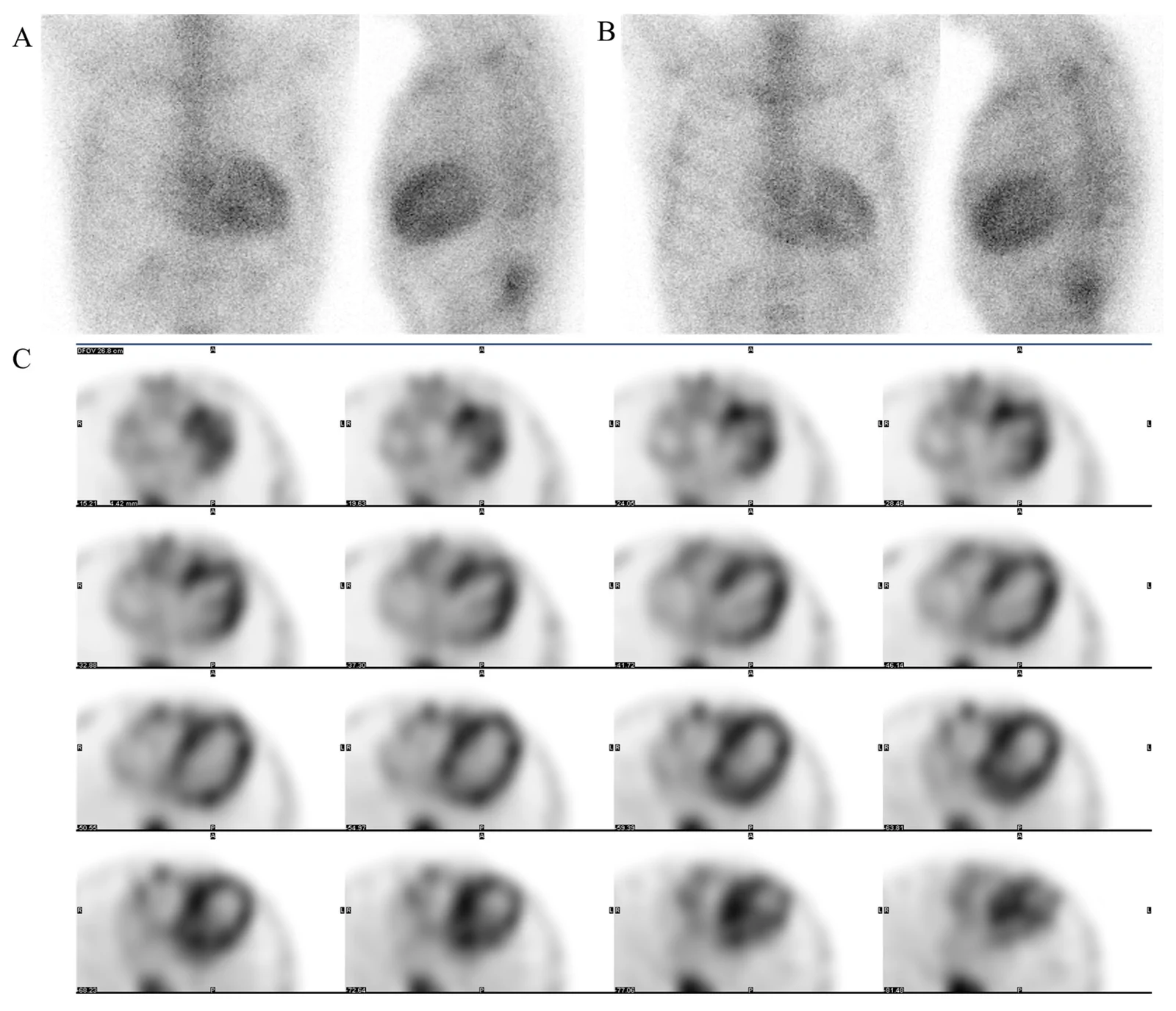
Technetium-99m pyrophosphate (^99m^Tc-PYP) scintigraphy in a patient. A thoracic scan in the anterior and sagittal views performed 1.5 (**A**)/3 (**B**) hours after administration of 99m-technetium pyrophosphate demonstrates grade 3 myocardial tracer uptake, with a heart-to-contralateral lung ratio > 1.5. There is intense radiotracer accumulation in the myocardium. (**C**) SPECT short-axis tomographic slices from base to apex reveal diffuse, homogeneous myocardial tracer retention with relative apical sparing, characteristic of transthyretin amyloid cardiomyopathy. No significant blood pool activity is noted on delayed imaging.

**Figure 5:**
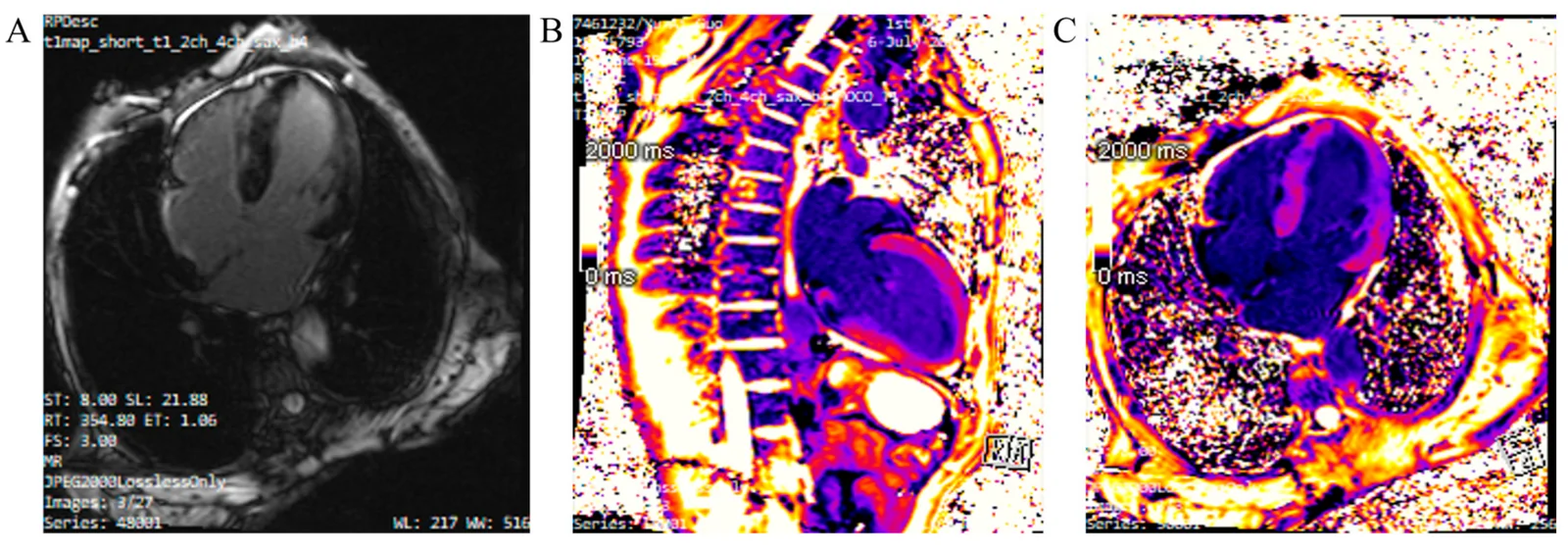
The cardiac magnetic resonance imaging (CMR) late gadolinium enhancement (LGE) pattern (**A**) and T1 native mapping (**B**,**C**) indicate cardiac amyloidosis.

## Data Availability

All data generated or analyzed during this study are included in this article. Further enquiries can be directed to the corresponding author.

## Supplementary Materials

The following supporting information can be downloaded at https://www.mdpi.com/article/10.3390/jcdd13100523/s1. Figure S1: X-ray crystallographic structural model of TTR in its tetrameric state (PDB code 1F41). TTR has four polypeptide chains. The p.Gly67Val variant, which replaces glycine with valine at residue 67, is highlighted in purple for clarity; Table S1: Dynamic changes in the ECG; Table S2: BNP 0–100 pg/mL; High-Sensitivity Troponin I (hs-cTnI) 0–0.0175 μg/L; Creatinine 57–111 μmol/L; eGFR > 90 mL/min; Table S3: IgG 7.23–16.85 g/L; IgA 0.69–3.82 g/L; IgM 0.63–2.77 g/L; C4 0.10–0.40 g/L; C3 0.79–1.52 g/L; Serum Kap 598–1329 mg/dL; Serum Lam 280–665 mg/dL; Serum KAP/LAM 1.30–3.00; Urine Kap 0.0–15.0 mg/dL; Urine Lam 0.0–15.0 mg/dL; Urine KAP/LAM 1.30–3.00; Table S4: Previously reported cases of ATTR-CA carrying the p.Gly67Val (legacy nomenclature: G47V, Gly47Val, G67V). F, Female; M, Male; PN, polyneuropathy; AN, autonomic neuropathy; H, cardiac involvement; FAP, familial amyloid polyneuropathy; NR, not reported.

## Abbreviations

The following abbreviations are used in this manuscript:

CA	Cardiac amyloidosis
ATTR-CA	Transthyretin cardiac amyloidosis
AL-CA	Immunoglobulin light chain cardiac amyloidosis
^99m^Tc-PYP	Technetium-99m pyrophosphate
QALY	Quality-adjusted life year
ECG	Electrocardiogram
LAH	Left anterior hemiblock
TTE	transthoracic echocardiogram
EF	Ejection fraction
IVST	Interventricular septal thickening
IgG	Immunoglobulins G
IgA	Immunoglobulins A
IgM	Immunoglobulins M
C4	Complement 4
C3	Complement 3
AVB	Atrioventricular block
CMR	Cardiac magnetic resonance imaging
LGE	Late gadolinium enhancement
CLBBB	Complete left bundle branch block
SSS	Sick sinus syndrome

## References

[1] Griffin J.M., Rosenblum H., Maurer M.S. (2021). Pathophysiology and Therapeutic Approaches to Cardiac Amyloidosis. Circ. Res..

[2] Ando Y., Coelho T., Berk J.L., Cruz M.W., Ericzon B.G., Ikeda S., Lewis W.D., Obici L., Planté-Bordeneuve V., Rapezzi C. (2013). Guideline of transthyretin-related hereditary amyloidosis for clinicians. Orphanet J. Rare Dis..

[3] Brown D., Vera-Llonch M., Perez J.T.O., Reddy S.R., Chang E., Tarbox M.H., Nativi-Nicolau J., Fine N. (2021). Use of commercial claims data to estimate transthyretin-amyloid cardiomyopathy prevalence and incidence in the US. JACC.

[4] Peters A.E., Solomon N., Chiswell K., Fonarow G.C., Khouri M.G., Baylor L., Alvir J., Bruno M., Huda A., Allen L.A. (2023). Transthyretin amyloid cardiomyopathy among patients hospitalized for heart failure and performance of an adapted wild-type ATTR-CM machine learning model: Findings from GWTG-HF. Am. Heart J..

[5] García-Pavía P., García-Pinilla J.M., Lozano-Bahamonde A., Yun S., García-Quintana A., Gavira-Gómez J.J., Aibar-Arregui M., Barge-Caballero G., Núñez Villota J., Bernal L. (2025). Prevalence of transthyretin cardiac amyloidosis in patients with heart failure with preserved ejection fraction: The PRACTICA study. Rev. Esp. Cardiol. (Engl. Ed.).

[6] Benson M.D., Teague S.D., Kovacs R., Feigenbaum H., Jung J., Kincaid J.C. (2011). Rate of progression of transthyretin amyloidosis. Am. J. Cardiol..

[7] Joury A., Faaborg-Andersen C., Quintana R.A., daSilva-deAbreu A., Nativi-Nicolau J. (2023). Diagnostic Tools for Cardiac Amyloidosis: A Pragmatic Comparison of Pathology, Imaging and Laboratories. Curr. Probl. Cardiol..

[8] Castano A., Haq M., Narotsky D.L., Goldsmith J., Weinberg R.L., Morgenstern R., Pozniakoff T., Ruberg F.L., Miller E.J., Berk J.L. (2016). Multicenter Study of Planar Technetium 99m Pyrophosphate Cardiac Imaging: Predicting Survival for Patients With ATTR Cardiac Amyloidosis. JAMA Cardiol..

[9] Castiglione V., Aimo A., Vergaro G. (2024). Cost-effectiveness of transthyretin cardiac amyloidosis screening and treatment: A Dilemma for the clinician. Int. J. Cardiol..

[10] Lau A.T.C., DiDomenico R.J., Kim K. (2024). Cost-effectiveness of systematic screening and treatment of transthyretin amyloid cardiomyopathy (ATTR-CM) in patients with heart failure with preserved ejection fraction (HFpEF) in United States. Int. J. Cardiol..

[11] Saraiva M.J. (1995). Transthyretin mutations in health and disease. Hum. Mutat..

[12] Fontana M., Banypersad S.M., Treibel T.A., Abdel-Gadir A., Maestrini V., Lane T., Gilbertson J.A., Hutt D.F., Lachmann H.J., Whelan C.J. (2015). Differential Myocyte Responses in Patients with Cardiac Transthyretin Amyloidosis and Light-Chain Amyloidosis: A Cardiac MR Imaging Study. Radiology.

[13] Asakura K., Yanai S., Nakamura S., Kawaski K., Eizuka M., Ishida K., Sugai T., Ueda M., Yamashita T., Ando Y. (2016). Endoscopic Findings of Small-Bowel Lesions in Familial Amyloid Polyneuropathy: A Case Report. Medicine.

[14] Gillmore J.D., Maurer M.S., Falk R.H., Merlini G., Damy T., Dispenzieri A., Wechalekar A.D., Berk J.L., Quarta C.C., Grogan M. (2016). Nonbiopsy Diagnosis of Cardiac Transthyretin Amyloidosis. Circulation.

[15] He X., Wang M., Sun J., Yu Z., Hu X., Liu Y., Lin X. (2024). Characterization of Transthyretin Mutation G47V Associated with Hereditary Cardiac Amyloidosis. Cardiology.

[16] Low S.C., Md Sari N.A., Tan C.Y., Ahmad-Annuar A., Wong K.T., Law W.C., Sim R.S., Lin K.P., Shahrizaila N., Goh K.J. (2021). Hereditary transthyretin amyloidosis in multi-ethnic Malaysians. Neuromuscul. Disord..

[17] González-Moreno J., Dispenzieri A., Grogan M., Coelho T., Tournev I., Waddington-Cruz M., Wixner J., Diemberger I., Garcia-Pavia P., Chapman D. (2024). Clinical and Genotype Characteristics and Symptom Migration in Patients With Mixed Phenotype Transthyretin Amyloidosis from the Transthyretin Amyloidosis Outcomes Survey. Cardiol. Ther..

[18] Booth D.R.S., Soutar A.K., Hawkins P.N., Reilly M., Harding A., Pepys M.B., Kisilevsky R., Benson M.D., Frangione B., Gauldie J., Muckle T.J., Young I.D. (1993). Three new amyloidogenic transthyretin gene mutations: Advantages of direct sequencing. Amyloid and Amyloidosis.

[19] Suenaga G., Ikeda T., Masuda T., Motokawa H., Yamashita T., Takamatsu K., Misumi Y., Ueda M., Matsui H., Senju S. (2017). Inflammatory state exists in familial amyloid polyneuropathy that may be triggered by mutated transthyretin. Sci. Rep..

[20] Rowczenio D., Quarta C.C., Fontana M., Whelan C.J., Martinez-Naharro A., Trojer H., Baginska A., Ferguson S.M., Gilbertson J., Rezk T. (2019). Analysis of the TTR gene in the investigation of amyloidosis: A 25-year single UK center experience. Hum. Mutat..

[21] Du K., Li F., Wang H., Miao Y., Lv H., Zhang W., Wang Z., Yuan Y., Meng L. (2021). Hereditary transthyretin amyloidosis in mainland China: A unicentric retrospective study. Ann. Clin. Transl. Neurol..

[22] Ferlini A., Obici L., Manzati E., Biadi O., Tarantino E., Conigli P., Merlini G., D’Alessandro M., Mazzaferro V., Tassinari C.A. (2000). Mutation and transcription analysis of transthyretin gene in Italian families with hereditary amyloidosis: A putative novel hot spot’ in codon 47. Clin. Genet..

[23] Pelo E., Da Prato L., Ciaccheri M., Castelli G., Gori F., Pizzi A., Torricelli F., Marconi G. (2002). Familial amyloid polyneuropathy with genetic anticipation associated to a gly47glu transthyretin variant in an Italian kindred. Amyloid.

[24] Tsampras T., Antonopoulos A.S., Kourti F.E., Tsioufis K., Vlachopoulos C. (2026). QTc interval prolongation as a marker of disease stage in transthyretin cardiac amyloidosis. Clin. Res. Cardiol..

[25] Orini M., Graham A.J., Martinez-Naharro A., Andrews C.M., de Marvao A., Statton B., Cook S.A., O’Regan D.P., Hawkins P.N., Rudy Y. (2019). Noninvasive Mapping of the Electrophysiological Substrate in Cardiac Amyloidosis and Its Relationship to Structural Abnormalities. J. Am. Heart Assoc..

[26] Nativi-Nicolau J., Judge D.P., Hoffman J.E., Gundapaneni B., Keohane D., Sultan M.B., Grogan M. (2021). Natural history and progression of transthyretin amyloid cardiomyopathy: Insights from ATTR-ACT. ESC Heart Fail..

[27] Maurer M.S., Schwartz J.H., Gundapaneni B., Elliott P.M., Merlini G., Waddington-Cruz M., Kristen A.V., Grogan M., Witteles R., Damy T. (2018). Tafamidis Treatment for Patients with Transthyretin Amyloid Cardiomyopathy. N. Engl. J. Med..

[28] Nativi-Nicolau J., Sarswat N., Fajardo J., Finkel M., Abdulsattar Y., Castaño A., Klein L., Haddad-Angulo A. (2021). Best Practices in Specialized Amyloidosis Centers in the United States: A Survey of Cardiologists, Nurses, Patients, and Patient Advocates. Clin. Med. Insights Cardiol..

[29] Gillmore J.D., Damy T., Fontana M., Hutchinson M., Lachmann H.J., Martinez-Naharro A., Quarta C.C., Rezk T., Whelan C.J., Gonzalez-Lopez E. (2018). A new staging system for cardiac transthyretin amyloidosis. Eur. Heart J..

