# Supplementary material for: Delayed Diagnosis of Hereditary Transthyretin Cardiac Amyloidosis Presenting with Autonomic Neuropathy: A Case Report
Source: J Cardiovasc Dev Dis. 2026 Oct 16;13(10):523. doi: 10.3390/jcdd13100523 (PMC13705157; doi:10.3390/jcdd13100523)
Supplement: Supplementary file 1 [file jcdd-13-00523-s001.zip › jcdd-4587079-supplementary.pdf]

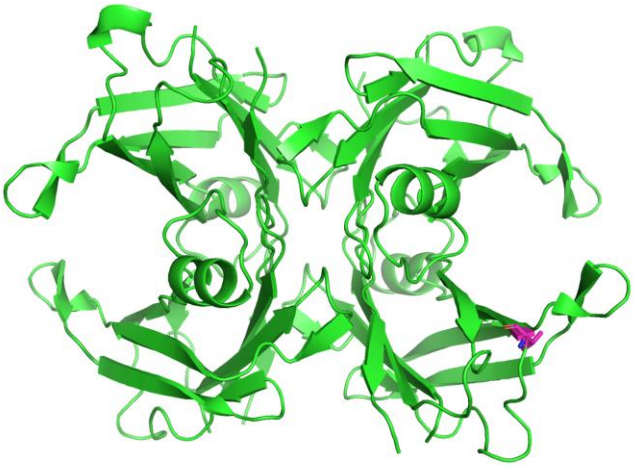

Figure S1. X-ray crystallographic structural model of TTR in its tetrameric state (PDB code 1F41). TTR has four polypeptide chains. The p. Gly67Val variant, which replaces glycine with valine at residue 67, is highlighted in purple for clarity.

Table S1. Dynamic changes in the ECG.

Date	Rhythm	PR interval (mm)	QRS duration (mm)	QTc (mm)	Main findings
20230827	69	188	108	416	Left anterior hemiblock and V1-V3 abnormal Q waves
20250918	71	172	120	479	-
20241223	60	222	126	476	First-degree atrioventricular block
20250331	50	210	136	456	Sinus Bradycardia
20250921	59	194	152	555	Complete left bundle branch block
20251201	47	176	152	493	-
20251202	60	248	142	480	Dual-chamber paced rhythm

Table S2: BNP 0–100 pg/mL; High-Sensitivity Troponin I (hs-cTnI) 0–0.0175 μg/L; Creatinine 57–111 μmol/L; eGFR > 90 mL/min;

Date	BNP (pg/mL)	High-Sensitivity Troponin I (μg/L)	Creatinine (μmol/L)	eGFR (ml/min)
20230827	115	0.0327	66	97
20250331	690	-	51	109
20250404	302		65	98
20250704	313.9	0.0449	71	94
20250921	272.1	0.0583	73	93
20251201	592	0.0419	75	92
20251203	745.3	0.2023	69	95
20260409	103.7	-	-	
20260508	-	-	77	91
20260608	123.4	-	-	
20260910	121.7	0.0194	75	92

Table S3. IgG 7.23–16.85 g/L; IgA 0.69–3.82 g/L; IgM 0.63–2.77 g/L; C4 0.10–0.40 g/L; C3 0.79–1.52 g/L; Serum Kap 598–1329 mg/dL; Serum Lam 280–665 mg/dL; Serum KAP/LAM 1.30–3.00; Urine Kap 0.0–15.0 mg/dL; Urine Lam 0.0–15.0 mg/dL; Urine KAP/LAM 1.30–3.00;

	20230827	20250402
IgG(g/L)	15.5 (-)	11.3 (-)
IgA(g/L)	1.58 (-)	0.98 (-)
IgM(g/L)	0.83 (-)	0.52 (↓)
C4(g/L)	0.23 (-)	0.15 (-)
C3(g/L)	0.58 (↓)	0.49 (↓)
Serum Kap(mg/dL)	-	884 (-)
Serum Lam(mg/dL)	-	529 (-)
Serum KAP/LAM	-	1.67
Urine Kap(mg/dL)	-	1.9 (-)
Urine Lam(mg/dL)	-	5 (-)
Urine KAP/LAM	-	0.38 (↓)
Serum immunofixation electrophoresis	-	(-)

Table S4. Previously reported cases of ATTR-CA carrying the p.Gly67Val (legacy nomenclature: G47V, Gly47Val, G67V). F, Female; M, Male; PN, polyneuropathy; AN, autonomic neuropathy; H, cardiac involvement; FAP, familial amyloid polyneuropathy; NR, not reported.

No.	Reference (first author, year)	Ethnicity / Region	No. of patients (sex)	Age at onset (y)	Phenotype	Initial Symptoms	Remarks
1	Booth et al., 1993 [18]	Sri Lankan	Kindred (n not stated)	NR	PN, AN, H	NR	First report; identified by direct sequencing
2	Asakura et al., 2016 [13]	Japanese	1 (F)	71	PN, AN (refractory diarrhea), H	AN (refractory diarrhea)	FAP with small-bowel lesions on endoscopy
3	Suenaga et al., 2017 [19]	Japanese	2 (F)	69	PN, AN, H	PN (Sensory disorder)	66 non-liver transplantation FAP patients
				71	AN, PN, H	AN	
4	Rowczenio et al., 2019 [20]	UK	7	NR	NR	NR	25-year UK National Amyloidosis Centre series (G67V in 7 of 724 mutation-positive cases)
5	Low et al., 2021 [16]	Malaysian	1 (F)	51	PN, AN, H	PN (Sensory disorder)	Phenotypic discordance between siblings (Gly47Val)
			1 (M)	60	PN, AN,	PN (Sensory disorder)	
6	Du et al., 2021 [21]	Chinese	1 (M)	62	PN, AN, H	PN (sensory disorder)	No family history (Gly47Val in 1 of 54 mutation-positive cases)
7	He et al., 2024 [15]	Chinese	1 (M)	48	H (conduction system disease)	conduction system	First biochemical/biophysical characterization; no PN/CTS/GI
